# Is Imaging Time Between two Tc 99m DMSA Scans Sufficient for Reporting as Renal Parenchymal Scarring? Healed Parenchymal Renal Defect After 6 Years

**DOI:** 10.4274/Mirt.22

**Published:** 2013-04-05

**Authors:** Erdem Sürücü, Yusuf Demir, Meral Torun Bayram, Salih Kavukçu, Hatice Durak

**Affiliations:** 1 Dokuz Eylül university, School of Medicine, Department of Nuclear medicine, İzmir, Turkey; 2 Dokuz Eylül university, School of Medicine, Departments of Pediatrics, İzmir, Turkey

**Keywords:** Kidney cortex necrosis, technetium Tc-99m dimercaptosuccinic acid, urinary tract infections

## Abstract

We aimed to report a healed renal parenchymal defect after 6 years in a 9-year-old girl who was being followed for recurrent urinary tract infection (UTI). The first UTI was at the age of two. She was being followed with ultrasonography, urine analysis and urine culture since the first UTI. Technetium-99m dimercaptosuccinic acid (DMSA) scintigraphy was repeated four times up to the present day. She had a renal parenchymal defect reported as parenchymal scarring, which healed 6 years after the first DMSA scintigraphy.

**Conflict of interest:**None declared.

## CASE REPORTS

**Figure 1:**
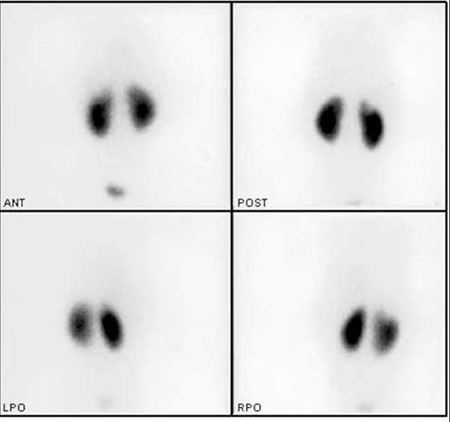
At the age of 2.5, anterior, posterior and oblique projections ofthe Tc 99m DMSA scan show a hypoactive area in the superior-lateral ofthe right kidney more prominent in the posterior image

**Figure 2:**
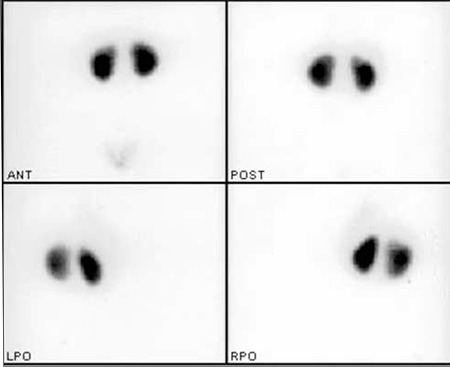
At the age of 3, Tc 99m DMSA scan 6 months later shows thatthe same hypoactive area in the superior-lateral of the right kidney is stillseen in the posterior image

A 9-year-old girl was referred to our nuclear medicine department for the differential diagnosis of acute pyelonephritis (APN) and renal parenchymal scarring. Vesicoureteral reflux (VUR) was diagnosed when she was at the age of 18 months. At admission of the first Technetium-99m dimercaptosuccinic acid (DMSA) scan, she did not have any symptoms, the last urine analysis and urine culture which was 1.5 months ago, were normal. First DMSA scintigraphy was performed when she was at the age of 2.5. A hypoactive area in the superior-lateral of the right kidney was reported (Figure 1) and repeating the scintigraphy after 6 months was recommended to differentiate acute pyelonephritis from renal scarring. Differential kidney functions were 50% for the right kidney and 50% for the left kidney. Ultrasonography (USG) was performed to look for any pathology in the superior-lateral part of the right kidney such as a cyst, kidney calculus or dilatation of pelvicalyceal system. However, no pathology was detected. After 6 months, at the age of 3, DMSA scintigraphy was repeated. The hypoactive area described in the previous scan was slightly decreased, but it was still present at the same location (Figure 2). The differential functions were the same as the previous scan. Before the second DMSA scan, the last urine analysis and urine culture, which was 1.5 months ago, were normal. It was again suggested to repeat the DMSA scintigraphy 6 months later for a final decision.

**Figure 3:**
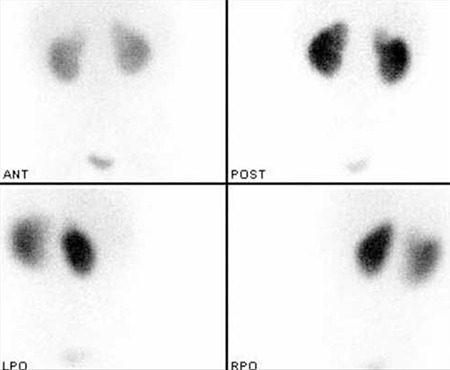
At the age of 5, Tc 99m DMSA scan two years after the secondscan reveals that the same hypoactive area in the superior-lateral of theright kidney is persistent and it was reported as renal parencymal scarring.There is also hypoactivity on the upper pole of the left kidney

She was lost for follow up for 2 years and finally she was again referr ed to our clinic for a follow up DMSA scintigraphy at the age of 5. USG was repeated and the findings were similar with the previous study. Also, there was hypoactivity on the upper pole of the left kidney. However, it was similar to previous study. Before the third DMSA scan, the last urine analysis and urine culture, which was 2 weeks ago, were normal. However, she had still bilateral vesicoureteral reflux. There was still a hypoactive area in the superior-lateral of the right kidney and finally this area was reported as renal parenchymal scarring (Figure 3). The differential functions were 49 % for the right kidney and 51 % for the left kidney.

**Figure 4:**
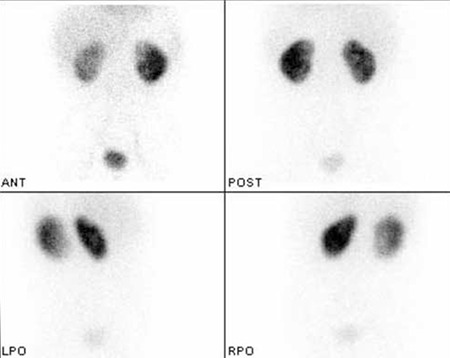
At the age of 9, 4th Tc 99m DMSA scan shows the superior-lateralof the right kidney is near normal in the posterior image, however thereis a mild hypoactive area that can be noticed in the anterior image

Two years after this third DMSA scan, the patient underwent bilateral subureteric injection for persistent vesicoureteral reflux. Evaluation of the therapy outcome for bilateral subureteric injection, she underwent direct vesicoureteral scintigraphy and vesicoureteral reflux was demonstrated in the right kidney. She could not be followed regularly for the next four years and she was again referred to our clinic to evaluate the status of right kidney 4 years after the third DMSA scan at the age of 9. E. Coli was seen in the last urine culture (5 days ago) and there was nitrite and white blood cells in the urine analysis. However she was afebrile, without any symptoms or leukocytosis. It was seen that the hypoactive area in the superior-lateral of the right kidney has almost disappeared (Figure 4). The differential functions were 39 % for the right kidney and 61 % for the left kidney.

## DISCUSSION

UTI is one of the most common important clinical problems in childhood, which occurs in 4–8% of infants with fever (1).No-specific symptoms in the children can differentiate lower UTI and acute pyelonephritis. It is estimated that 60–65% of patients with febrile UTI may have APN (2). UTI can affect the renal parenchyma and the children with renal parenchymal involvement are at risk of permanent renal damage that may lead to renal scarring, hypertension, complications during pregnancy and renal failure (3). Renal scarring is present in 8-40% of patients after an episode of APN (4,5). Experimental (6,7,8) and clinical studies (9,10) showed that the DMSA scintigraphy is probably the most sensitive method for diagnosing and localizing APN. Focal or diffuse reduction of DMSA accumulation develops in APN, however the border of renal parenchyma is protected (11,12). DMSA scan is more sensitive and specific than USG and Intravenous Pyelogram (IVP) for detecting renal parenchymal scarring and follow-up (13). Because DMSA accumulates in functional kidney cells, in contrast to APN, functional cortex is lost in renal parenchymal scarring. Since the recovery time and the severity of the infection can change from child to child, DMSA scan is recommended to be repeated at least 6 months after acute infection (14).

The degree of infection is also important for the choice of treatment and prognosis. DMSA scintigraphy can also help to differentiate the level of the infection. In addition, DMSA scintigraphy is more sensitive than intravenous urography for detecting renal scarring (15,16).

DMSA scintigraphy still seems to be the only imaging tool that can show the progression of the acute damage of renal parenchyma after pyelonephritis and following the development of renal scarring. The proposed repeating time of the scintigraphy for differentiating acute pyelonephritis from renal scarring is variable; the guidelines suggest that it should be at least 6 months (14). If renal damage is still being reported after 6 months, renal parenchymal scarring should be considered. In our case, the persistent hypoactive area described in three successive DMSA scintigraphies suggests renal parenchymal scarring.

One reason of this appearance might be recurrent APN. The patient might have had APN during all of the three DMSA scintigraphies and the hypoactive areas in the kidney might be due to repeated APN. Moreover, there were no clinical infection symptoms such as fever and blood test abnormalities such as increased CRP level, leukocytosis during these DMSA scans and there were not any microorganisms in the urine culture before the DMSA scans. In patients with APN, if there is a reduced Tc-99m DMSA uptake in the scan, clinical infection symptoms or blood test abnormalities should be noticed (17). So, asymptomatic or culture-negative infections can be seen very rarely when there is an abnormal DMSA scan.

Previous studies have shown that the most of the renal scarring was caused by VUR (18). On the other hand, recent studies suggested that scarring may often occur in the absence of VUR (19) and it has been argued that renal scarring may be independent from the presence or absence of VUR (20). Although there was bilateral grade III vesicoureteral reflux in the voiding cystogram before the third DMSA scan, only right kidney had renal parenchymal scarring.

Another point for this case might be related with renal dysplasia, which is characterized by the formation of structural defects of the nephron and collecting ducts accompanied with fibrosis, like scar tissue (21). While the hypoactive area in the superior-lateral of the right kidney improved with growing up of the child, 10% difference in the differential functions of the kidneys in the last DMSA scan occurred. As the body grows up, dysplasic kidney may not have sufficient functional tissue. This might explain the differences in the differential functions between the kidneys. But contrarily, there was no pathology in two of the USG during the follow-up. Furthermore, USG might have missed out the pathology due to lesion size.

In conclusion, DMSA scintigraphy is still the most sensitive imaging tool to evaluate renal parenchymal scarring and the follow-up. The persistent hypoactive areas in three DMSA scans might be related to persistent infections. Furthermore, because same hypoactivity was seen in three DMSA scans, it was reported as renal parenchymal scarring. Although the optimal time between two Tc-99m DMSA scans for reporting as renal parenchymal scarring is 3-6 months, our patient’s renal defect seems to be healed 6 years after the first DMSA scan or at all the times the patient was scanned, the patient had persistent infections.
